# Construction and Evaluation of a High-Frequency Hearing Loss Screening Tool for Community Residents

**DOI:** 10.3390/ijerph182312311

**Published:** 2021-11-23

**Authors:** Yi Wang, Chengyin Ye, Dahui Wang, Chenhui Li, Shichang Wang, Jinmei Li, Jinghua Wu, Xiaozhen Wang, Liangwen Xu

**Affiliations:** Department of Prevention Medicine, Hangzhou Normal University, Hangzhou 311121, China; wangyi2@stu.hznu.edu.cn (Y.W.); yechengyin@hznu.edu.cn (C.Y.); 20120051@hznu.edu.cn (D.W.); 2018111012012@stu.hznu.edu.cn (C.L.); 2018111012039@stu.hznu.edu.cn (S.W.); lijinmei@stu.hznu.edu.cn (J.L.); wujinghua@stu.hznu.edu.cn (J.W.); 2020112012102@stu.hznu.edu.cn (X.W.)

**Keywords:** high-frequency hearing loss, lasso regression, risk assessment model, risk factor, community residents

## Abstract

Early screening and detection of individuals at high risk of high-frequency hearing loss and identification of risk factors are critical to reduce the prevalence at community level. However, unlike those for individuals facing occupational auditory hazards, a limited number of hearing loss screening models have been developed for community residents. Therefore, this study used lasso regression with 10-fold cross-validation for feature selection and model construction on 38 questionnaire-based variables of 4010 subjects and applied the model to training and testing cohorts to obtain a risk score. The model achieved an area under the curve (AUC) of 0.844 in the model validation stage and individuals’ risk scores were subsequently stratified into low-, medium-, and high-risk categories. A total of 92.79% (1094/1179) of subjects in the high-risk category were confirmed to have hearing loss by audiometry test, which was 3.7 times higher than that in the low-risk group (25.18%, 457/1815). Half of the key indicators were related to modifiable contexts, and they were identified as significantly associated with the incident hearing loss. These results demonstrated that the developed model would be feasible to identify residents at high risk of hearing loss via regular community-level health examinations and detecting individualized risk factors, and eventually provide precision interventions.

## 1. Introduction

Hearing loss is the fourth leading cause of disability worldwide [1]. According to the World Health Organization (WHO), approximately 466 million people are estimated to be living with disabling hearing loss status, and 1.1 billion young individuals are at a risk of hearing loss. In general, hearing loss will seriously affect an individual’s working ability, social function, and mental health, resulting in a large amount of social resource consumption and a heavy social burden.

Etiologies of hearing loss are multifactorial, and the common risk factors include genetic variation, noise exposure, ototoxic drugs, aging, and lifestyle factors [2]. There is growing evidence showing many modifiable risk factors for hearing loss and, if these were eliminated, half the cases of hearing loss could be prevented [3]. Therefore, early screening and detection of individuals with modifiable risk factors for hearing loss are the first steps towards the reduction of its prevalence.

Hearing loss usually starts from high-frequency hearing issues and then degenerates to language frequency disability; high-frequency hearing loss screening is the key to early detection of hearing issues [4]. The WHO has recommended that countries should develop and initiate monitoring and screening programs to facilitate early detection of individuals at high risk of hearing loss. The current gold standard for the diagnosis of hearing impairment is pure-tone audiometry, which requires expensive audiology equipment and trained professionals, so large-scale hearing testing is not feasible at the community level [5]. A potentially desirable and actionable solution is to divide individuals into different risk groups using simple predictive tools, determine risk factors, and identify the high-risk segments of the population for implementation of subsequent lifestyle interventions or administration of medical treatments, ultimately preventing them from progressing to actual hearing loss status.

Many researchers from relevant study fields have developed screening tools for hearing-impaired listeners, including scales and technology-based hearing screening tools [6,7]. Most of the questionnaires used currently were developed for elder people to measure the functional status of hearing deficit and assess the severe outcomes caused by this deficit. For example, the Hearing Handicap Inventory in the Elderly (HHIE) [8] and Korean Evaluation Scale for Hearing Handicap (KESHH) [9] are representative of such questionnaires, which are specifically designed for detecting emotional and social problems induced by hearing loss for the elderly hearing-impaired patients. Meanwhile, another questionnaire, Self-assessment for Hearing Screening of the Elderly (SHSE) [10], focused on general issues related to hearing loss, such as distracting conditions, fast-rate speech, and working memory. These scales did not include hearing loss risk factors and were not designed to screen younger adults at risk of hearing loss.

For the purpose of screening or prediction, most previous hearing loss prediction models typically focused on professional workers who have occupational noise exposures, or individuals afflicted with a sudden sensorineural hearing loss (SSHL). There are several prediction models for evaluating such hearing loss risk. One model was developed for the prediction of hearing loss in individuals facing occupational auditory hazards, and it showed a discriminatory accuracy of 78.2% [11]. Another study adopted machine learning algorithms to develop a prediction model to predict prognosis of SSHL, attaining an area under the curve (AUC) of 0.84 [12]. Unlike the definition of hearing loss in our study, SSHL is a common otologic emergency, defined as an abrupt onset of sensorineural hearing loss (≥30 dB) affecting at least three consecutive frequencies within 72 hours. Either occupational risk factors, such as intensive or complex industrial noise exposure for a long duration, or risk factors related to SSHL, are quite different from those hearing loss risk factors prevalent in community residential area.

Therefore, the aim of this study was to establish a high-frequency hearing loss risk prediction model for adults residing in the community, by using data from 4010 residents of Zhejiang province; to apply the built model to the training and testing cohorts and obtain risk scores that describe an individual’s probability of receiving a diagnosis of high-frequency hearing loss; and to stratify residents into low-, medium-, and high-risk categories based on these risk scores. We further aimed to identify modifiable risk factors for hearing loss, and to provide guidance for screening of hearing loss and intervention of high-risk categories.

## 2. Materials and Methods

### 2.1. Participants

**Figure A1:**
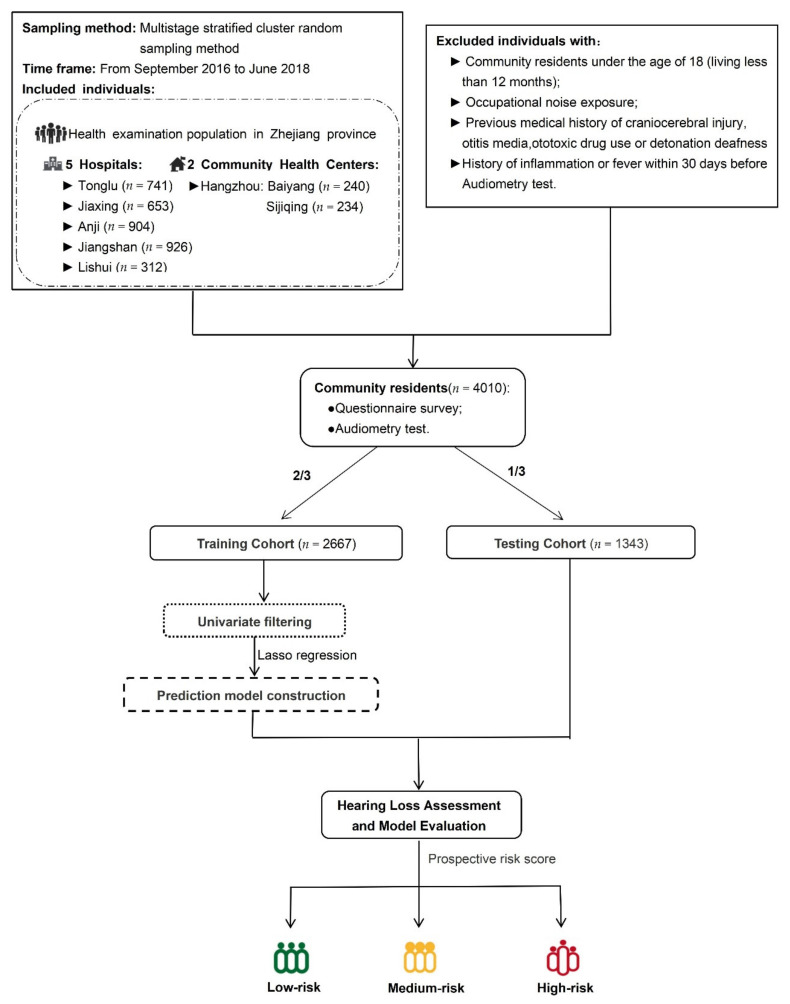
A workflow diagram depicting high-frequency hearing loss model construction and evaluation.

The multistage, stratified cluster random sampling method was used to examine the health of residents in Zhejiang province at five hospitals and two community health centers from September 2016 to June 2018. After providing informed consent, 4010 residents participated in the survey and completed the questionnaire survey and audiometry test. The inclusion criteria are shown in Figure A1. This study was approved by the institutional review board of Hangzhou Normal University (No.2017LL107), and all personal privacy information was well protected.

### 2.2. Audiometry Test

All pure-tone air-conduction hearing thresholds were measured by professional medical staff using audiometers (AT235; Interacoustics, Assens, Denmark) and TDH-39 headphones (Telephonic Corporation, Farmingdale, New York, NY, USA) in a well-ventilated listening chamber with a background noise <30 dB(A). Subjects were recommended to stay away from noisy environments for more than 12 h before the audiometry test in order to improve accuracy. In the intensity range of −10 to 110 dB, pure-tone air conduction hearing thresholds were tested in both ears of the participants at frequencies of 0.125, 0.25, 0.5, 1, 2, 3, 4, 6, and 8 kHz [13]. Participants who did not respond at least once in the audiometry test were considered as nonrespondents. To measure the reliability of participants’ responses, tests were performed twice on the 1 kHz frequency in each ear. If the results differed by more than 10 dB, the response was considered unreliable and then tests would be performed again. The diagnosis criteria of high-frequency hearing loss were an average hearing threshold of the standard frequency band (3, 4, 6, and 8 kHz) of hearing in a poor ear higher than 25dB (A) [14,15].

### 2.3. Questionnaire Survey

The questionnaire survey and audiometry test were conducted on the same day. The original questionnaire was first evaluated and revised through expert consultation. After the formal questionnaire was generated, a pilot survey involving 926 participants was first conducted to verify the questionnaire. Before the implement of the pilot survey, all investigators had been well trained to ensure the quality of data collection. As a result, they were able to provide accurate explanations of both items and options in a face-to-face interview, thereby minimizing the respondent’s bias. During the pilot survey, the developed questionnaire reached a Cronbach α coefficient of 0.753, and a KMO value of 0.794.

The questionnaire included six parts: (1) demographics and social determinants (age, gender, marital status, education, average monthly household income, familial disease, and self-perceived hearing status); (2) symptom histories (tinnitus history, inner ear pain history, and aural fullness history); (3) disease conditions (current hypertension, hyperlipidemia, diabetes mellitus, high cholesterol, arteriosclerosis, anemia, migraine, coronary heart disease, acute and chronic otitis media, and tumors); (4) behavioral factors (smoking, secondhand smoking, alcohol consumption, bedtime, hours of sleep, earphone frequency, electronic device volume, daily fruit and vegetable intaking, and exercise frequency); (5) environmental exposure (workplace noise exposure, living noise exposure, work stress, and life stress); and (6) hearing cognitive parameters (pay attention to your hearing, pay attention to hearing protection, regular hearing check, and hearing protection skills). The details of the questionnaire items and the answers given by the respondents are shown in Table S2.

Previous research has identified a broad range of risk factors for hearing loss which can be classified into three groups [3], including nonmodifiable, partly modifiable, and fully modifiable risk factors. Nonmodifiable risk factors were defined as factors which cannot be altered through intervention, such as age and gender. Partly modifiable risk factors were defined as factors which cannot be easily altered through intervention (e.g., social determinants, symptom histories, and disease conditions). Conversely, fully modifiable risk factors can be changed, controlled, or repaired by intervention, such as behavioral factors, environmental exposures, and hearing cognitive parameters (Table S1).

It should be noted that symptom histories (e.g., tinnitus history and inner ear pain history) were measured by the frequency of their self-reported occurrence within the past year, while disease histories (e.g., current hypertension, and hyperlipidemia) were determined based on participants’ self-reported current or historical physician diagnosis. In terms of behavioral conditions, most variables were measured as current or past-1-year conditions, while smoking/drinking status were measured as none or little smoking/drinking, former smoking/drinking, or current smoking/drinking (Table S1). In addition, the noise exposure was measured as the subjective feeling of the exposure within the past year, so if the participant felt that the sound was too loud and uncomfortable, then he/she was considered to be exposed to the noise. It is worth mentioning that in our study, it was assumed that most of the measured current or past year’s behavioral and lifestyle conditions, environmental exposure, and hearing cognition factors were a relatively stable long-term state and would not change over time.

### 2.4. Statistical Analysis

#### 2.4.1. Features Selection

We used Epidata V.3.1 (The Epidata Association, Odense, Denmark) to enter the survey data and to check and correct errors. Before feature selection, in order to ensure the authenticity of the data, the samples corresponding to the missing data were directly deleted. The cases were identified according to audiometry test results. Chi-squared test was adopted to evaluate the difference between various covariates between cases and controls, where all reported probability values were two-tailed, and a *p*-value of <0.05 was considered for statistical significance. After applying this criterion, 36 out of 38 features were selected (Figure A1).

#### 2.4.2. Model Construction and Evaluation

The cohort (*n* = 4010) was randomly divided into a training cohort (*n* = 2667) and a testing cohort (*n* = 1343) at a ratio of 2/3:1/3; 36 features were selected, and the least absolute shrinkage and selection operator (lasso) regression with 10-fold cross-validation and penalty was used to construct a hearing loss prediction model by “glmnet” R software package. Lasso regression is a widely used machine learning algorithm; compared with traditional logistic regression, it uses a penalty term, which can actively select impactful parameters from a large set of potentially multicollinear variables in the regression, helping to reduce prediction errors [16].

In the process of model evaluation, high-frequency hearing loss risk score was calculated from the lasso regression in the cohort. We ranked all the individuals (*n* = 4010) from low risk to high risk according to the risk score, and divided them into three groups: low-, medium-, and high-risk group. Performance of the model was investigated within each risk category in terms of positive predictive value (PPV). All statistical analyses were performed using R V.4.0. (Figure A1).

## 3. Results

### 3.1. Demographic Characteristics and Variable Selection

The cohort comprised 4010 residents, 55.7% (2232/4010) of whom were diagnosed with high-frequency hearing loss. Among these 2232 cases, 55.5% (1238/2232) were male and 26.3% (586/2232) received junior high school education. The major diseases included current hypertension (32.0%, 714/2232) and diabetes mellitus (6.9%, 154/2232). In terms of lifestyles, 25.0% (558/2232) of patients with hearing loss smoked, 21.1% (470/2232) consumed alcohol, 45.7% (1019/2232) barely exercised, and 63.2% (1410/2232) rarely worked in a noise exposure environment. The remaining descriptive statistics for all other predictors are available in Table S2. A total of 36 out of 38 features were selected after univariate analysis.

### 3.2. Model Performance

**Figure 1:**
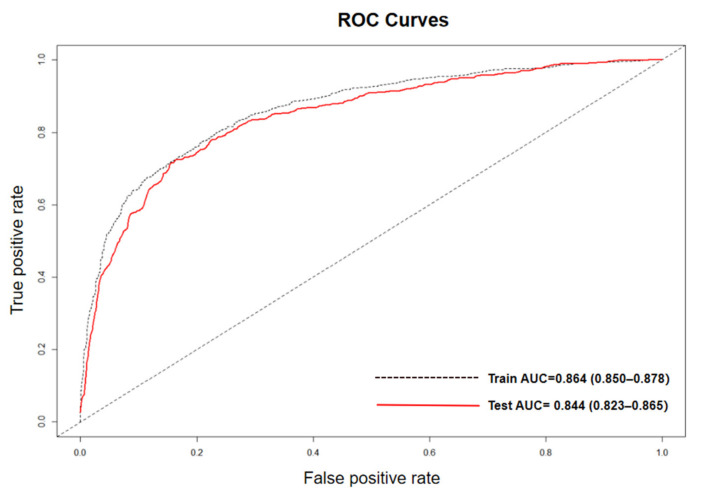
Receiver operating characteristic (ROC) curves applied on the train cohort and the test cohort, respectively.

Application of the lasso regression to the data showed that our prediction model achieved a fitted AUC of 0.864 (95% confidence interval (CI) 0.850–0.878) in the training cohort and a predicted AUC of 0.844 (95% CI 0.823–0.865) in the testing cohort (Figure 1). Based on the calculated risk score, the cohort was divided into three risk groups (Table 1): low-risk group (score 0–0.50), medium-risk group (score 0.50–0.80), and high-risk group (score 0.80–1.00). A total of 1815 individuals were included in the low-risk group, and 25.18% of them (457/1815) were affected by high-frequency hearing loss. In contrast, among the 1179 individuals included in the high-risk group, more than 92.79% (1094/1179) were diagnosed with high-frequency hearing loss (Table 1).

### 3.3. Significant Features

**Figure 2:**
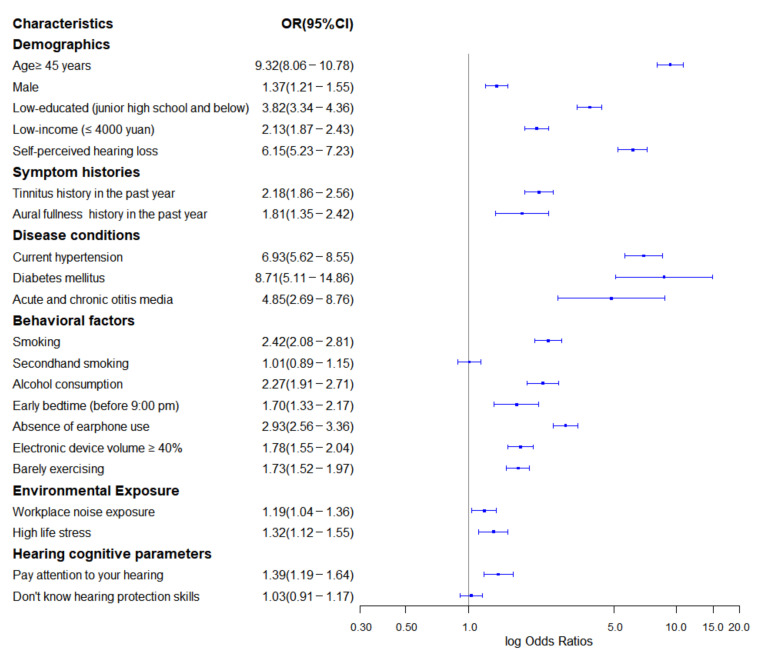
Forest plot of high-frequency hearing loss odds ratios and their 95% confidence intervals.

A total of 22 impactful features were identified as the final predictors of the model, including 20 predictors that were related to modifiable contexts and two nonmodifiable predictors. The estimated coefficients are listed in Table S3. These predictors and their adjusted odds ratios (ORs) or coefficients between cases and controls as well as their 95% CIs were derived from the original cohort (Figure 2). Age ≥ 45 years, self-perceived hearing loss, and low-educated populations were recognized as the demographic characteristics that were strongly associated with hearing loss, with ORs of 9.32, 6.15, and 3.82, respectively. Diabetes mellitus, current hypertension, and otitis media were the most relevant diseases, with ORs of 8.71, 6.93, and 4.85, respectively. In lifestyle category, smoking (OR = 2.42), alcohol consumption (OR = 2.27), and electronic device volume ≥ 40% (OR = 1.78) were identified as powerful predictors.

### 3.4. Distribution Patterns Stratified According to Risk Categories

#### 3.4.1. Lifestyle-Related Feature Difference

**Figure 3:**
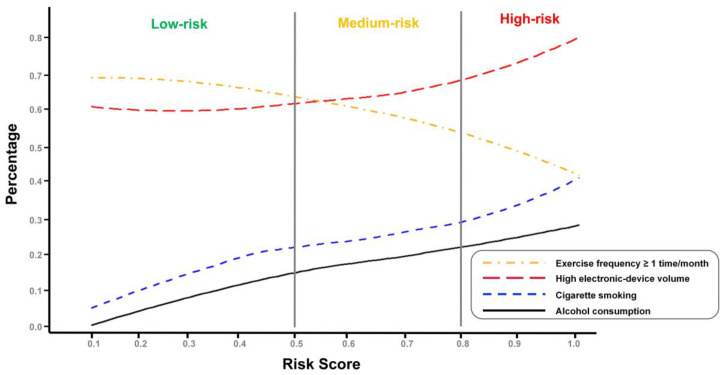
The percentage curves of behavior factors across the identified three risk categories.

To further explore the distribution of captured lifestyles across the three risk categories, we grouped individuals across the spectrum of risk scores and calculated the prevalence of certain lifestyles under each risk bin. As shown in Figure 3, the proportion of individuals who consumed alcohol, smoked, and used high electronic device volume increased significantly along with an increase in the risk score, whereas the proportion of people who exercised >1 time/month decreased dramatically. Specifically, 70.0% (1271/1815) of individuals in the low-risk group had exercise frequency >1 time/month, and the frequency dropped to 46.4% (547/1179) in the high-risk group. A total of 62.8% (1139/1815) of individuals in the low-risk group used high electronic device volume, and the frequency increased to 81.0% (955/1179) in the high-risk group. A total of 29.0% (342/1179) in the high-risk group drink alcohol, which was 2.9 times higher than that in the low-risk group (10.0%, 182/1815). Similarly, 40.4% (476/1179) of individuals in the high-risk group were smokers which is 2.5 times that of the low-risk group 15.9% (288/1815).

#### 3.4.2. Age-Related Feature Difference

**Figure 4:**
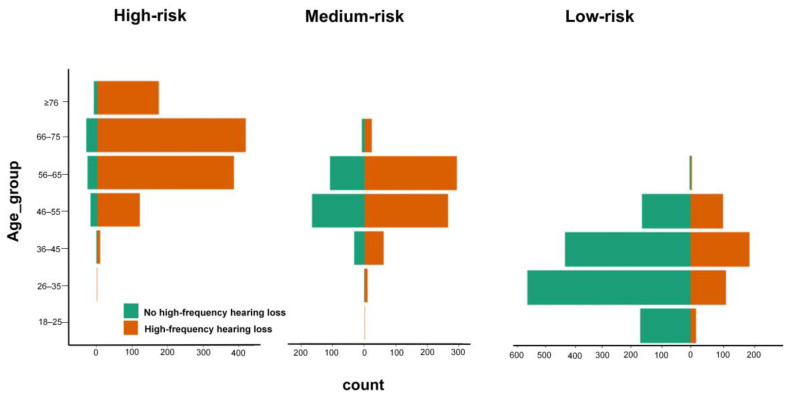
The number of age subgroups across the identified three risk groups. Age groups (years): 18–25, 26–35, 36–45, 46–55, 56–65, 66–75, ≥76.

Age was recognized as an impactful demographic feature in our high-frequency hearing loss prediction model. Among seven distinct age groups included in this study, young individuals mainly constituted the low-risk group, whereas older individuals were concentrated in the high-risk group (Figure 4). Regarding age, composition of the high-risk group was as follows: 66–75 years, 35.03% (413/1179); 56–65 years, 32.23% (380/1179). In the medium-risk group, 30.12% (306/1016) of patients with high-frequency hearing loss were 56–65 years old. On the contrary, only 25.18% (457/1815) of individuals in the low-risk group had high-frequency hearing loss, most of whom were 36–45 years old (44.42%, 203/457). In addition, we further focused on people over 65 years of age in our study and evaluated the discriminatory ability of our developed model in this subgroup. As a result, our prediction model attained a sensitivity of 94.15% (193/205) in the validation set for identifying hearing loss patients in the elderly population. The distribution of age groups across the three risk categories are show in Table S4.

#### 3.4.3. Disease-Related Feature Difference

**Figure 5:**
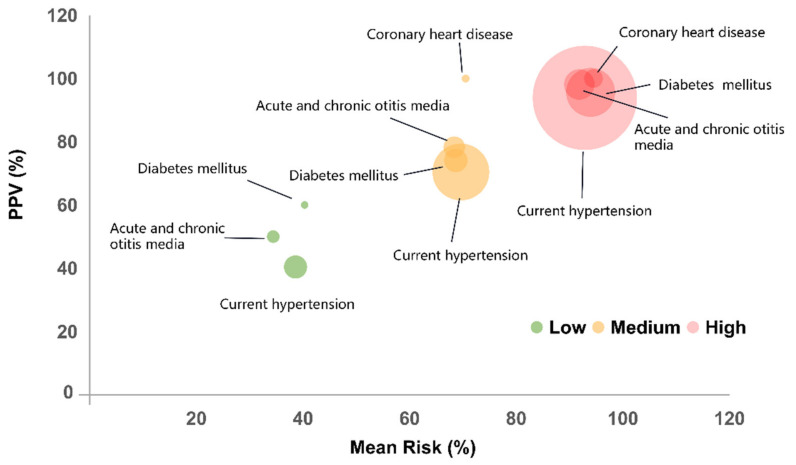
Disease history subgroup’s average risk against the PPV. The balls were formed by 4 disease subgroups under the low-risk (green balls), medium- risk (yellow balls), and high-risk (red balls) categories, respectively. The centers of the circles are the mean risk and PPV values. The ball size indicates the proportion of the disease subgroup under this risk category. The 4 diseases were current hypertension, diabetes mellitus, coronary heart disease, and acute and chronic otitis media.

The individuals diagnosed with four common diseases (i.e., current hypertension, diabetes mellitus, coronary heart disease, and acute and chronic otitis media) showed a dramatic increase in hearing loss risk score from the range corresponding to the low-risk group to that corresponding to the high-risk group (Figure 5). More than 57.42% (677/1179) of individuals in the high-risk group had at least one of these four diseases, and ≥ 14.47% (147/1179) of individuals in the high-risk group had at least two of these four diseases. On the contrary, only 3.53% (64/1815) and 0.11% (2/1815) of individuals in the low-risk group had least one or two of these six diseases. Specifically, hypertension and diabetes mellitus affected 52.67% (621/1179) and 11.62% (137/1179) of the high-risk population but had limited impact on the low-risk population, with only 2.59% (47/1815) and 0.28% (5/1815) being diagnosed with these two diseases, respectively.

## 4. Discussion

### 4.1. Summary of Main Findings

The high-frequency hearing loss screening model developed in this study achieved an AUC of 0.844 in the model validation stage, indicating that it has a good discriminatory ability and could be potentially applied to community residents living in southeast China, using their self-reported predictors. Using this prediction model, we classified the population into high-, medium-, and low-risk categories. The PPV of the high-risk group was 92.79%, which was 3.7 times higher than that of the low-risk group (25.18%), and half of the predictors were related to modifiable context. It is believed that the developed model could facilitate initial screening and help identify individuals at a high risk of hearing loss for the implementation of precision intervention.

### 4.2. Interpretation of Meaningful Risk Predictors and Its Implications for Prevention and Early Intervention

#### 4.2.1. Social Determinants and Lifestyles

Social determinants are associated with health from various perspectives [17]. Understanding differences among patients in terms of their social determinants will enable decision-makers and health care systems to prioritize screening of the population at the highest risk and, consequently, facilitate the development of targeted interventions that are essential for the improvement of health and reduction of health disparities [18]. Several studies have shown that hearing loss risk is associated with socioeconomic factors such as education [19,20] and income level [21]. Consistent with these studies, our study found that individuals with low education and income levels had relatively higher risk of hearing loss than those with high education and income levels. Possible mechanisms include the fact that lower income was usually linked with poor access to, utilization of, and quality of, health care, and was then correlated with poorer health status [22]. Lower education was a marker of unhealthy lifestyle attributes (e.g., second-hand smoking and alcohol consumption) that were included in our prediction model [23].

Smoking, alcohol consumption, and exercise habits, which are shaped by social and economic factors, are impactful behavioral drivers of hearing loss [24]. Smoking could have a short-term contribution to hearing loss risk, because the direct and indirect transmission of endogenous reactive oxygen species caused by smoking can affect cochlear function. In addition, smoking may also increase blood viscosity, resulting in reduced blood flow to the cochlea and, eventually, hearing loss [25]. Notably, a prospective study among women revealed that hearing loss risk may diminish with greater duration of smoking cessation [26]. Meanwhile, our study observed that excessive volume of electronic devices can also lead to hearing loss. Previous studies have shown that high electronic device volume caused damage to the cochlear hair cells and can lead to tinnitus, disability of the sound afferent, and increased susceptibility to age-related hearing loss [27,28], so the studies recommended that the earphone volume be set at less than 50% of the maximum volume [29]. Although alcohol consumption was identified as a risk factor of hearing loss in our study, controversial results have been reported elsewhere; while some studies suggest that alcohol consumption may increase the risk of hearing loss [30], others suggest that it may reduce the risk [31]. Therefore, large and well-designed cohort studies are required to verify this. Similar to our study, a cohort study observed that physical activity was related to better hearing, because regular exercise could reduce age-related stria capillary loss, thereby delaying the progress of hearing loss [32,33].

In addition, high stress in daily life was recognized as a predictor of hearing loss in our screening model, which may also have interacted with material and interpersonal social determinants, triggering unhealthy lifestyle choices such as excessive alcohol consumption and visceral obesity [34], and eventually inducing hearing issues through biological pathways of neuroendocrine, neuroimmune, and epigenetic responses [35].

#### 4.2.2. Age and Gender

Age and gender were nonmodifiable predictors in our prediction model. Hearing decline along with aging is generally attributed to progressive peripheral degeneration, including the degeneration of cochlear sensory hair cells, stria vascularis, and spiral ganglion neurons leading to the disability of sound afferent [36]. Our prediction model confirmed that, besides the elderly population (≥65 years), the middle-aged (46–65 years) population also showed high prevalence of high-frequency hearing loss, which may easily be overlooked in community settings. Our study found that males were more severely affected by hearing loss than females in terms of earlier age of onset and more rapid progression of the disease. Male and female individuals may have distinct genetic and cumulative social risk factors, which are closely related with the difference in hearing loss [37].

#### 4.2.3. Multiple Diseases and Symptom Histories

In our study, hypertension was a predictor, and it may increase the risk of hearing loss by reducing blood supply to the stria vascularis, which is in the lateral cochlear wall and is responsible for sending auditory signals from the cochlea to the central nervous system. Vascular supply to the stria vascularis is derived from terminal arteries with no collateral supply. Therefore, the stria vascularis is particularly sensitive to events that compromise vascular supply, with animal studies showing reduced end cochlear potential and hearing loss occurring promptly after an anoxic event [38]. Diabetes mellitus is a common systemic metabolic disease, and it may result in cochlear microangiopathy, degeneration of the stria vascularis, and loss of cochlear outer hair cells. All these diseases have been shown in prospective studies to increase the risk of hearing loss, suggesting that preventing hypertension and diabetes could potentially reduce the burden of hearing loss [39]. Thus, early detection and timely intervention may have public health value for hearing loss prevention.

Symptom histories, such as tinnitus history and aural fullness history, were also recognized as modifiable indicators of hearing loss in our model. Tinnitus, the perceived sensation of a sound that has no external source [40], could be regarded as a symptom that may be caused by lifestyle factors. Tinnitus can arise from pathological changes along the entire auditory pathway [41]. Research has shown that persistent tinnitus was associated with substantially higher risk of 3-year hearing threshold elevation [42]. Therefore, timely and effective treatment for tinnitus, such as psychological treatment and early behavioral lifestyle interventions, may have a positive effect on hearing loss prevention.

### 4.3. Comparison with Other Studies

To date, prediction models for high-frequency hearing loss in the general population are still lacking. Unlike our prediction model, most existing hearing loss screening tools (e.g., HHIE and uHear) only focus on evaluating the severe outcomes and functional status of hearing deficit and fail to identify individualized risk factors of hearing loss. It was reported that under different circumstances, the sensitivity of HHIE (the instrument for elderly) could range from 24% to 100% [43,44]. On the other hand, when only focusing on the elderly population, our prediction model attained a sensitivity of 94.15% (193/205) in the validation set for identifying hearing loss patients, indicating relatively good performance. Moreover, the hearing loss prediction model developed for the professional worker or individuals afflicted with an SSHL cannot be directly applied to general population living in communities, who may have quite different risk factors from those professional workers, and the model may be subject to low performance. It is also worth mentioning that our model is not only suitable for the elderly, but also designed for young adults, and our model can be utilized to identify individualized risk factors and provide clues for personalized intervention.

### 4.4. Strengths and Limitations

At present, hearing loss prediction models developed for community residents is limited; it is believed that our screening model can help improve the delivery of health care services at multiple levels. First, compared with existing tools, it has the advantages of low cost, ease of implementation, and no need for supporting equipment. Second, the model can stratify the population based on the obtained risk score of hearing loss, which can help to target high-risk populations and facilitate decision-making and implementation of interventions. Third, the model, as a screening tool, can help primary care physicians identify individuals who should be referred to hospitals for audiometry tests to facilitate the diagnosis of hearing loss (high risk vs. low risk). Most importantly, the model can identify essential and critical modifiable risk factors for each community resident. On this basis, healthcare workers could design personalized health education and promotion programs to improve individuals’ self-consciousness capabilities in relation to high-frequency hearing loss.

Our study also has several limitations. First, self-reported noise exposure information was obtained, which could cause potential bias and misclassification. Second, the model’s performance should be further validated in an independent cohort, ideally consisting of a population from different areas of China to guarantee the model’s generalizability to a larger number of people. Third, the risk factors involved in this study may not be comprehensive enough, and the built model’s screening ability may be further improved after the collection of more valuable predictors.

## 5. Conclusions

This study developed and validated a risk prediction model to identify high-risk groups of community residents with high-frequency hearing loss. The model consisted of simple questionnaire survey items and showed the risk score of individual factors. The risk screening model can easily identify people at high risk of hearing loss and the unique risk factors of each community resident. On this basis, medical workers could design personalized health education and promotion programs to improve individuals’ self-consciousness and self-care capabilities in relation to high-frequency hearing loss.

## Figures and Tables

**Table 1:** The performance of high-frequency hearing loss risk prediction model in the cohort.

Risk Category	Low	Medium	High	Total
Intervals	[0,0.50]	[0.50,0.80]	[0.80,1.00]	
Total, *n*	1815	1016	1179	4010
Case, *n*	457	681	1094	2232
PPV, %	25.18	67.03	92.79	

## Data Availability

Not applicable.

## Supplementary Materials

The following are available online at https://www.mdpi.com/article/10.3390/ijerph182312311/s1, Table S1: Summary of the nonmodifiable, partly- and fully modifiable risk factors included in the questionnaire., Table S2: Characteristics of high-frequency hearing loss cases and controls, obtained from the questionnaire., Table S3: The estimated coefficients in the lasso regression., Table S4: Distribution of impactful risk predictors across the three risk categories.
