# Supplementary material for: Construction and Evaluation of a High-Frequency Hearing Loss Screening Tool for Community Residents
Source: Int J Environ Res Public Health. 2021 Nov 23;18(23):12311. doi: 10.3390/ijerph182312311 (PMC8657277; doi:10.3390/ijerph182312311)
Supplement: Supplementary file 1 [file ijerph-18-12311-s001.zip › ijerph-1420768-supplementary.pdf]

Table S1. Summary of the non-modifiable, partly- and fully modifiable risk factors included in the questionnaire.

Category	Characteristic	Predictors	Time evolution	Definitions
Non-modifiable	Demographics and social determinants	Age	Current	—
		Gender	Current	—
Partly Modifiable		Marital status	Current	—
		Education	Current	Low-educated (junior high school and below)
		Average monthly household income	Current	Low-income (≤ 4000 yuan)
		Familial disease	Current	—
		Self-perceived hearing status	Current	Self-perceived hearing loss (petty loss and above)
	Symptom histories	Tinnitus history	Within the last year	—
		Inner ear pain history	Within the last year	—
		Aural fullness history	Within the last year	—
	Disease conditions	Current hypertension	Current or in the past	Anamnestic diagnosis
		Hyperlipidemia	Current or in the past	Anamnestic diagnosis
		Diabetes mellitus	Current or in the past	Anamnestic diagnosis
		High cholesterol	Current or in the past	Anamnestic diagnosis
		Arteriosclerosis	Current or in the past	Anamnestic diagnosis
		Anemia	Current or in the past	Anamnestic diagnosis
		Migraine	Current or in the past	Anamnestic diagnosis
		Coronary heart disease	Current or in the past	Anamnestic diagnosis
		Acute and chronic otitis media	Current or in the past	Anamnestic diagnosis
		Tumors	Current or in the past	Anamnestic diagnosis

Fully Modifiable	Behavioral factors	Smoking	Current or in the past	1) none or little (smoking less than one cigarette a day on average for less than 1 year), 2) former (cessation of smoking since the past 6 months or more),3) current (smoking at least one cigarette a day for more than 1 year)
		Secondhand smoking	Current or in the past	—
		Alcohol consumption	Current or in the past	1) none or little (less than once per week, each time the liquor does not exceed 150ml, and the beer does not exceed 500ml), 2) former (abstinence for more than 6 months), 3) current (at least once a week and lasts for at least half a year or more)
		Bedtime	Within the last year	—
		Hours of sleep	Within the last year	—
		Earphone frequency	Within the last year	—
		Electronic device volume	Within the last year	—
		Daily fruit and vegetable intaking	Within the last year	—
		Exercise frequency	Within the last year	—
	Environmental exposure	Workplace noise exposure	Within the last year	Workplace noise exposure was defined as the participant's exposure to noise in the workplace at least once a week.
		Living noise exposure	Within the last year	Living noise exposure was defined as participants' exposure to noise living environment at least once a week (e.g., loud music or power tools).
		Work stress	Within the last year	—
		Life stress	Within the last year	High life stress (relatively large and very large)
	Hearing cognitive parameters	Pay attention to your hearing	Within the last year	—
		Pay attention to hearing protection	Within the last year	—

	Regular hearing check	Within the last year	—
	Hearing protection skills	Within the last year	—

Table S2. Characteristics of high-frequency hearing loss cases and controls, obtained from the questionnaire.

Characteristic	Study cohort (N=4010)	Control n (%) (N=1778)	Case n (%) (N=2232)	χ^2	*p*-value
**Demographics and social determinants**					
Age（years）				1277.001	< 0.001
18-25	195(4.9)	176(9.9)	19(0.9)		
26-35	702(17.5)	570(32.1)	132(5.9)		
36-45	751(18.7)	476(26.8)	275(12.3)		
46-55	871(21.7)	363(20.4)	508(22.8)		
56-65	834(20.8)	145(8.2)	689(30.9)		
66-75	476(11.9)	39(2.2)	437(19.6)		
≥ 76	181(4.5)	9(0.5)	172(7.7)		
Gender				23.95	< 0.001
Male	2086(52.0)	848(47.7)	1238(55.5)		
Female	1924(48.0)	930(52.3)	994(44.5)		
Marital status				232.357	< 0.001
Unmarried	316(07.9)	264(14.8)	52(2.3)		
Married	3580(89.3)	1484(83.5)	2096(93.9)		
Divorce	62(1.5)	24(1.3)	38(1.7)		
Widowed	52(1.3)	6(0.3)	46(2.1)		
Education				548.564	< 0.001
Primary schools and below	552(13.8)	68(3.8)	484(21.7)		
Junior high school	826(20.6)	240(13.5)	586(26.3)		
High school or technical secondary school	1000(24.9)	435(24.5)	565(25.3)		
Junior college or undergraduate	1590(39.7)	1009(56.7)	581(26.0)		
Postgraduate and above	42(1.0)	26(1.5)	16(0.7)		
Average monthly household income (yuan)				225.934	< 0.001
≤ 2000	600(15.0)	106(6.0)	494(22.1)		
2001-4000	995(24.8)	426(24.0)	569(25.5)		
4001-6000	1531(38.2)	782(44.0)	749(33.6)		
6001-8000	674(16.8)	352(19.8)	322(14.4)		
> 8000	210(5.2)	112(6.3)	98(4.4)		
Familial disease				55.136	< 0.001
No	3726(92.9)	1712(96.3)	2014(90.2)		
Yes	284(7.1)	66(3.7)	218(9.8)		
Self-perceived hearing status				567.807	< 0.001
Good	2722(67.9)	1550(87.2)	1172(52.5)		
Petty loss	946(23.6)	204(11.5)	742(33.2)		
Moderate loss	274(6.8)	22(1.2)	252(11.3)		
Severe loss	56(1.4)	1(0.1)	55(2.5)		
Extreme loss	12(0.3)	1(0.1)	11(0.5)		

**Symptom histories**					
Tinnitus history within the past year				127.979	< 0.001
No	3125(77.9)	1512(85.0)	1613(72.3)		
Monthly	568(14.2)	212(11.9)	356(15.9)		
Weekly	183(4.6)	33(1.9)	150(6.7)		
Everyday	134(3.3)	21(1.2)	113(5.1)		
Inner ear pain history within the past year				50.749	< 0.001
No	3809(95.0)	1735(97.6)	2074(92.9)		
Monthly	146(3.6)	38(2.1)	108(4.8)		
Weekly	47(1.2)	3(0.2)	44(2.0)		
Everyday	8(0.2)	2(0.1)	6(0.3)		
Aural fullness history within the past year				18.683	< 0.001
No	3786(94.4)	1708(96.1)	2078(93.1)		
Monthly	185(4.6)	62(3.5)	123(5.5)		
Weekly	25(0.6)	5(0.3)	20(0.9)		
Everyday	14(0.3)	3(0.2)	11(0.5)		
**Disease conditions**					
Current hypertension				397.241	< 0.001
No	3183(79.4)	1665(93.6)	1518(68.0)		
Yes	827(20.6)	113(6.4)	714(32.0)		
Hyperlipidemia				55.806	< 0.001
No	3778(94.2)	1730(97.3)	2048(91.8)		
Yes	232(5.8)	48(2.7)	184(8.2)		
Diabetes mellitus				89.911	< 0.001
No	3841(95.8)	1763(99.2)	2078(93.1)		
Yes	169(4.2)	15(0.8)	154(6.9)		
High cholesterol				8.689	0.003
No	3907(97.4)	1747(98.3)	2160(96.8)		
Yes	103(2.6)	31(1.7)	72(3.2)		
Arteriosclerosis				11.811	0.001
No	3991(99.5)	1777(99.9)	2214(99.2)		
Yes	19(0.5)	1(0.1)	18(0.8)		
Anemia				0.002	0.964
No	3864(96.4)	1713(96.3)	2151(96.4)		
Yes	146(3.6)	65(3.7)	81(3.6)		
Migraine				3.929	0.047
No	3879(96.7)	1731(97.4)	2148(96.2)		
Yes	131(3.3)	47(2.6)	84(3.8)		
Coronary heart disease				18.427	< 0.001
No	3987(99.4)	1778(100.0)	2209(99.0)		
Yes	23(0.6)	0(0.0)	23(1.0)		
Acute and chronic otitis media				33.339	< 0.001
No	3920(97.8)	1765(99.3)	2155(96.6)		
Yes	90(2.2)	13(0.7)	77(3.4)		

Tumors				0.739	0.39
No	3995(99.6)	1773(99.7)	2222(99.6)		
Yes	15(0.4)	5(0.3)	10(0.4)		
**Behavioral factors**					
Smoking				156.89	< 0.001
None or very little	2954(73.7)	1471(82.7)	1483(66.4)		
Former	226(5.6)	35(2.0)	191(8.6)		
Current	830(20.7)	272(15.3)	558(25.0)		
Secondhand smoking				49.25	< 0.001
No	2166(54.0)	963(54.2)	1203(53.9)		
1-2 days a week	862(21.5)	446(25.1)	416(18.6)		
3-5 days a week	453(11.3)	197(11.1)	256(11.5)		
≥ 5 days a week	529(13.2)	172(9.7)	357(16.0)		
Alcohol consumption				88.07	< 0.001
None or very little	3282(81.8)	1569(88.2)	1713(76.7)		
Former	68(1.7)	19(1.1)	49(2.2)		
Current	660(16.5)	190(10.7)	470(21.1)		
Bedtime				126.758	< 0.001
< 21	308(7.7)	101(5.7)	207(9.3)		
21-23	2726(68.0)	1096(61.6)	1630(73.0)		
≥ 23	976(24.3)	581(32.7)	395(17.7)		
Hours of sleep				32.34	< 0.001
< 4 hours	27(0.7)	6(0.3)	21(0.9)		
4-6 hours	237(5.9)	77(4.3)	160(7.2)		
6-8 hours	2583(64.4)	1218(68.5)	1365(61.2)		
≥ 8 hours	1163(29.0)	477(26.8)	686(30.7)		
Earphone frequency				253.343	< 0.001
Never	2727(68.0)	980(55.1)	1747(78.3)		
Once a week	785(19.6)	462(26.0)	323(14.5)		
1-2 times a day	423(10.5)	283(15.9)	140(6.3)		
≥ 3 times a day	75(1.9)	53(3.0)	22(1.0)		
Electronic device volume				176.896	< 0.001
< 40 %	1224(30.5)	664(37.3)	560(25.1)		
40-80 %	2018(50.3)	928(52.2)	1090(48.8)		
≥ 80 %	768(19.2)	186(10.5)	582(26.1)		
Daily fruit and vegetable intaking				46.263	< 0.001
< 500g	1617(40.3)	612(34.4)	1005(45.0)		
≥ 500g	2393(59.7)	1166(65.6)	1227(55.0)		
Exercise frequency				111.523	< 0.001
Barely	1600(39.9)	581(32.7)	1019(45.7)		
1-3 times a month	780(19.5)	407(22.9)	373(16.7)		
1-2 times a week	650(16.2)	342(19.2)	308(13.8)		
3-4 times a week	369(9.2)	211(11.9)	158(7.1)		
> 4 times a week	611(15.2)	237(13.3)	374(16.8)		

**Environmental Exposure**					
Workplace noise exposure (categories)				17.147	< 0.001
None or very little	2603(64.9)	1193(67.1)	1410(63.2)		
At least once a week	947(23.6)	422(23.7)	525(23.5)		
At least once a day	460(11.5)	163(9.2)	297(13.3)		
Workplace noise exposure (binary)				6.697	0.01
No	2603(64.9)	1193(67.1)	1410(63.2)		
Yes	1407(35.1)	585(32.9)	822(36.8)		
Living noise exposure				12.073	0.002
None or very little	3088(77.0)	1412(79.4)	1676(75.1)		
At least once a week	612(15.3)	252(14.2)	360(16.1)		
At least once a day	310(7.7)	114(6.4)	196(8.8)		
Work stress				167.473	< 0.001
Very large	111(2.8)	61(3.4)	50(2.2)		
Relatively large	956(23.8)	400(22.5)	556(24.9)		
Common	1562(39.0)	808(45.4)	754(33.8)		
Relatively small	906(22.6)	419(23.6)	487(21.8)		
Very small	475(11.8)	90(5.1)	385(17.2)		
Life stress				136.028	< 0.001
Very large	106(2.6)	49(2.8)	57(2.6)		
Relatively large	650(16.2)	245(13.8)	405(18.1)		
Common	1717(42.8)	886(49.8)	831(37.2)		
Relatively small	1036(25.8)	479(26.9)	557(25.0)		
Very small	501(12.5)	119(6.7)	382(17.1)		
**Hearing cognitive parameters**					
Pay attention to your hearing				68.743	< 0.001
Don't care	732(18.3)	374(21.0)	358(16.0)		
Not much attention	1151(28.7)	496(27.9)	655(29.3)		
Common	1220(30.4)	565(31.8)	655(29.3)		
Attention	733(18.3)	313(17.6)	420(18.8)		
Very concerned	174(4.3)	30(1.7)	144(6.5)		
Pay attention to hearing protection				54.537	< 0.001
Don't care	824(20.5)	391(22.0)	433(19.4)		
Not much attention	1045(26.1)	458(25.8)	587(26.3)		
Common	1330(33.2)	629(35.4)	701(31.4)		
Attention	651(16.2)	271(15.2)	380(17.0)		
Very concerned	160(4.0)	29(1.6)	131(5.9)		
Regular hearing check				18.825	< 0.001
No need	570(14.2)	263(14.8)	307(13.8)		
Uncertain	1499(37.4)	613(34.5)	886(39.7)		
Needed	1680(41.9)	798(44.9)	882(39.5)		
Necessary	207(5.2)	87(4.9)	120(5.4)		
Very necessary	54(1.3)	17(1.0)	37(1.7)		
Hearing protection skills				55.838	< 0.001

don't know or know very little	2219(55.3)	976(54.9)	1243(55.7)
Understand	1192(29.7)	558(31.4)	634(28.4)
Know	238(5.9)	125(7.0)	113(5.1)
Familiar	186(4.6)	86(4.8)	100(4.5)
Very Familiar	175(4.4)	33(1.9)	142(6.4)

Table S3. The estimated coefficients in the Lasso regression.

Variable	β
Intercept	-2.912
Demographics and social determinants	
Age (years)	0.700
Gender	-0.199
Education	-0.266
Average monthly household income (yuan)	-0.033
Self-perceived hearing status	0.850
Symptom histories	
Tinnitus history within the past year	0.053
Aural fullness history within the past year	-0.070
Disease histories	
Current hypertension	0.583
Diabetes mellitus	0.276
Coronary heart disease	0.850
Acute and chronic otitis media	0.593
Behavioral factors	
Smoking	0.056
Secondhand smoking	0.034
Alcohol consumption	0.031
Bedtime	-0.021
Earphone frequency	0.008
Electronic device volume	0.092
Exercise frequency	-0.018
Environmental exposure	
Workplace noise exposure	0.070
Life stress	-0.066
Hearing cognitive parameters	
Pay attention to your hearing	0.066
Hearing protection skills	0.056

Table S4. Distribution of impactful risk predictors across the three risk categories.

Risk category	Low	Medium	High
Risk score intervals	[0,0.50]	[0.50,0.80]	[0.80,1.00]
**High-frequency hearing loss**	457	681	1094
**Demographics and social determinants**			
Age (years)			
18-25	3.9%	0.1%	0.0%
26-35	26.5%	1.5%	0.1%
36-45	44.4%	9.3%	0.8%
46-55	24.5%	40.7%	10.9%
56-65	0.7%	44.9%	34.7%
66-75	0.0%	3.5%	37.8%
≥ 76	0.0%	0.0%	15.7%
Gender			
Male	51.6%	50.4%	60.2%
Female	48.4%	49.6%	39.8%
Education			
Primary schools and below	0.9%	14.5%	34.8%
Junior high school	10.5%	30.1%	30.4%
High school or technical secondary school	23.6%	31.0%	22.5%
Junior college or Undergraduate	62.1%	24.1%	12.2%
Postgraduate and above	2.8%	0.3%	0.1%
Average monthly household income (yuan)			
≤ 2000	4.4%	19.5%	31.2%
2001-4000	16.6%	25.7%	29.1%
4001-6000	50.3%	34.1%	26.2%
6001-8000	22.1%	15.1%	10.8%
> 8000	6.6%	5.6%	2.7%
Self-perceived hearing status			
Good	90.4%	72.8%	24.0%
Petty loss	9.6%	25.3%	48.1%
Moderate loss	0.0%	1.8%	21.9%
Severe loss	0.0%	0.1%	4.9%
Extreme loss	0.0%	0.0%	1.0%
**Symptom histories**			
Tinnitus history within the past year			
No	90.2%	76.4%	62.2%
Monthly	7.0%	15.1%	20.2%
Weekly	1.8%	5.0%	9.9%
Everyday	1.1%	3.5%	7.7%
Aural fullness history within the past year			
No	96.9%	94.0%	91.0%
Monthly	2.6%	5.1%	6.9%
Weekly	0.4%	0.6%	1.3%

Everyday	0.0%	0.3%	0.8%
**Disease conditions**			
Current hypertension			
No	95.8%	83.6%	46.7%
Yes	4.2%	16.4%	53.3%
Diabetes mellitus			
No	99.3%	97.1%	88.0%
Yes	0.7%	2.9%	12.0%
Coronary heart disease			
No	100.0%	99.6%	98.2%
Yes	0.0%	0.4%	1.8%
Acute and chronic otitis media			
No	98.5%	97.4%	95.2%
Yes	1.5%	2.6%	4.8%
**Behavioral factors**			
Smoking			
None or very little	79.0%	70.0%	59.0%
Former	2.6%	4.8%	13.3%
Current	18.4%	25.1%	27.7%
Secondhand smoking			
No	54.9%	52.3%	54.5%
1-2 days a week	23.9%	19.7%	15.8%
3-5 days a week	10.3%	12.8%	11.2%
≥ 5 days a week	10.9%	15.3%	18.6%
Alcohol consumption			
None or very little	86.7%	79.7%	70.7%
Former	1.1%	1.3%	3.2%
Current	12.3%	18.9%	26.1%
Bedtime			
< 21	3.3%	7.3%	13.0%
21-23	70.7%	71.5%	75.0%
≥ 23	26.0%	21.1%	12.1%
Headphone frequency			
Never	50.3%	80.3%	88.7%
Once a week	34.1%	13.4%	6.9%
1-2 times a day	13.3%	5.4%	3.8%
≥ 3 times a day	2.2%	0.9%	0.5%
Electronic device volume			
< 40%	30.0%	31.4%	19.1%
40%-80%	62.8%	48.6%	43.1%
≥ 80%	7.2%	20.0%	37.8%
Exercise frequency			
Barely	31.7%	41.7%	53.9%
1-3 times a month	26.3%	18.1%	11.9%

1-2 times a week	22.3%	15.3%	9.3%
3-4 times a week	9.6%	6.9%	6.1%
> 4 times a week	10.1%	18.1%	18.7%
**Environmental exposure**			
Workplace noise exposure			
No	57.1%	69.2%	62.0%
Yes	42.9%	30.8%	38.0%
Life stress			
Very large	2.2%	3.2%	2.3%
Relatively large	21.4%	16.0%	18.1%
Common	51.0%	34.4%	33.3%
Relatively small	20.6%	28.2%	24.8%
Very small	4.8%	18.2%	21.6%
**Hearing cognitive parameters**			
Pay attention to your hearing			
Don't care	17.9%	14.1%	16.5%
Not much attention	27.8%	28.0%	30.8%
Common	37.2%	31.3%	24.9%
Attention	15.5%	20.4%	19.2%
Very concerned	1.5%	6.2%	8.7%
Hearing protection skills			
Don't know or know very little	53.6%	50.8%	59.6%
Understand	36.1%	32.3%	22.8%
Know	6.8%	5.1%	4.3%
Familiar	2.4%	5.1%	4.9%
Very Familiar	1.1%	6.6%	8.4%
